# Caloric vestibular stimulation induces vestibular circular vection even with a conflicting visual display presented in a virtual reality headset

**DOI:** 10.1177/20416695231168093

**Published:** 2023-04-20

**Authors:** Ramy Kirollos, Chris M. Herdman

**Affiliations:** Defence Research and Development Canada, Toronto Research Center, Toronto, Ontario, Canada; Visualization and Simulation Center, 6339 Carleton University, Ottawa, Ontario, Canada

**Keywords:** vestibular circular vection, visual-vestibular sensory integration, vection speed, caloric vestibular stimulation, VR headsets

## Abstract

This study explored visual-vestibular sensory integration when the vestibular system receives self-motion information using caloric irrigation. The objectives of this study were to (1) determine if measurable vestibular circular vection can be induced in healthy participants using caloric vestibular stimulation and (2) determine if a conflicting visual display could impact vestibular vection. In Experiment 1 (E1), participants had their eyes closed. Air caloric vestibular stimulation cooled the endolymph fluid of the horizontal semi-circular canal inducing vestibular circular vection. Participants reported vestibular circular vection with a potentiometer knob that measured circular vection direction, speed, and duration. In Experiment 2 (E2), participants viewed a stationary display in a virtual reality headset that did not signal self-motion while receiving caloric vestibular stimulation. This produced a visual-vestibular conflict. Participants indicated clockwise vection in the left ear and counter-clockwise vection in right ear in a significant proportion of trials in E1 and E2. Vection was significantly slower and shorter in E2 compared to E1. E2 results demonstrated that during visual-vestibular conflict, visual and vestibular cues are used to determine self-motion rather than one system overriding the other. These results are consistent with optimal cue integration hypothesis.

## Background

The objective of the current study was to examine visual-vestibular sensory integration to determine self-motion direction during conflict. This was done by using caloric vestibular stimulation (CVS) to produce measurable vestibular circular vection in Experiment 1 (E1) by stimulating the horizontal semi-circular canals (SCCs) with a current of monaural (i.e., in one ear) cold air. We examined how vestibular vection was influenced by a visual-vestibular conflict during CVS in Experiment 2 (E2).

Vection is commonly defined as the illusory experience of self-motion while an individual is stationary (Dichgans & Brandt, 1978). Linear vection refers to illusory self-translation. Circular vection is illusory self-motion about the yaw axis of rotation while the individual is stationary. Vection has traditionally been studied in the visual modality (Mach, 1875; Palmisano et al., 2015), however, auditory vection (Keshavarz et al., 2014; Riecke et al., 2008) and vestibular vection (Fischer & Wodak, 1924; Fitzpatrick et al., 2002; Fitzpatrick & Watson, 2015; St George et al., 2011) have also been examined (see Palmisano et al. (2015) for a review of definitions of vection). The study of vection can provide insight into the visual and vestibular sensory systems. In applied settings, the experience of vection is believed to be important to add realism in virtual environments such as those used for training, gaming, and entertainment (Weech et al., 2019).

The visual system can detect both constant velocity self-motion and acceleration. On the other hand, the vestibular system is optimized for detecting accelerations of the head in the linear and angular axes of motion (Howard, 1982). The SCCs in the bony labyrinth of the inner-ear detect angular acceleration of the head. There is a set of three SCCs in each ear: the horizontal, superior and posterior SCC. The horizontal SCCs detect yaw-axis rotation and are positioned at a 30° angle from the earth's horizontal (Baloh, 2003), the superior SCCs detect pitch-axis rotation and the posterior SCCs detects roll-axis rotation in each ear (Rabbitt, 2019). The superior and posterior SCC are both in the vertical plane relative to gravity but are orthogonally configured (Baloh, 2003). The orthogonal 3D configuration of the SCCs in each ear permits detection of head acceleration with six degrees of freedom (Rabbitt, 2019). The SCCs contain the cupular membrane in which hair cells are embedded within the endolymph fluid. When an individual's head rotates, the endolymph fluid shifts, causing bending of hair cells. The reciprocal configuration of the SCCs in each ear creates an excitatory response in one ear, and an inhibitory response in the other creating a distinct activation pattern that signals the direction of motion to the brain via the vestibular nerve in the 8^th^ cranial nerve (Bordoni et al., 2021). The otolith organs in the peripheral vestibular system primarily sense linear accelerations whereas the SCCs detect angular accelerations in the yaw pitch and roll axes. The fluid dynamic properties of the peripheral vestibular system make it sensitive to inertial motion but not constant motion. Therefore, the vestibular system cannot distinguish between moving at constant velocity and being stationary (Lishman & Lee, 1973).

Research on visual-vestibular sensory integration has yielded evidence supporting three hypotheses. Studies supporting visual dominance hypothesis have shown that the visual system will override the vestibular system to decide on self-motion. On this view, it has been shown that optic flow can strongly influence an individual's standing posture, even though the vestibular cues indicate that they are stationary (Berthoz et al., 1975; Lee & Lishman, 1975; Lishman & Lee, 1973; Warren, 1895). On the other hand, support for a vestibular dominance hypothesis has been found. For example, Butler et al. (2010) found that a person's perception of heading direction was more consistent with vestibular cues than with visual cues. Similarly, Harris et al. (2000) found that reports of traversed distances were more consistent with vestibular cues to motion than visual cues to motion.

The third hypothesis states that perceived motion is based on an optimal weighted average of visual and vestibular sensory inputs (Fetsch et al., 2010). Optimal cue integration hypothesis assumes that information from multiple sensory systems, in this case, visual and vestibular systems, are fused in a statistically optimal fashion to reduce perceptual uncertainties (Rohde et al., 2016). Researchers have shown that optimal cue integration hypothesis follows rules of Maximum Likelihood Estimation (MLE) (Clarke & Yuille, 1990; Ernst & Banks, 2002). In MLE, each sensory modality's estimates of a perceptual judgement are combined into a weighted average with others based on the precision of the individual sensory system's information to reduce perceptual uncertainty. According to optimal cue integration hypothesis, the modality (visual or vestibular) that senses the most reliable input provides the greatest weight in determining the perception of heading. “Reliability” is defined as the inverse of the variance of a cue over trials (Fetsch et al., 2010). Therefore, cue reliability is tied to its strength or the level to which the cue is compelling. The optimal cue integration hypothesis has been supported by studies examining visual-vestibular conflict during linear (Fetsch et al., 2010; Gu et al., 2008) and rotational (De Winkel et al., 2013; De Winkel et al., 2010; Juergens et al., 2016; Jürgens & Becker, 2011; Reymond et al., 2002; Telban & Cardullo, 2001) heading perception. Heading and vection are different: heading does not require self-motion perception to be experienced whereas vection does (Kirollos et al., 2017; Lepecq et al., 2006). Heading only requires that the observer is able to distinguish the direction of implied self-motion signaled by the optic flow pattern (Kirollos et al., 2017; Lepecq et al., 2006) (see Britton and Arshad (2019) for an alternative definition). Heading perception occurs much more quickly than vection.

Fetsch et al. (2009) investigated the impact of cue reliability on heading perception. Vestibular cues for motion were generated by having subjects (human and non-human primates) sit in a chair that moved linearly. Concurrent visual cues were provided with a visual display of moving dots that indicated self-motion. Cue reliability was varied by manipulating the percentage of dots in the visual display that moved together to signal the direction of motion.

In another study on optimal cue integration hypothesis, De Winkel et al. (2013) assessed heading perception by varying visual and vestibular cues while participants sat in a chair. In a visual-only condition, participants saw moving black and white stripes but the chair remained stationary. In a vestibular-only condition, the chair rotated but the visually presented stripes did not. In a combined condition, both the chair and the stripes moved congruently. De Winkel et al. found that the perception of heading direction was most influenced in the visual-only cue condition for five of the eight participants. The three remaining participants made heading judgements based on the cue combination condition. These findings demonstrate a deviation from optimal cue integration hypothesis as findings should have indicated that heading perception should have been influenced most by the visual-vestibular condition–the cue combination hypothesized to be the most reliable. Instead, participants used visual cues more frequently to decide heading even when visual-vestibular cues were provided. The results of this study demonstrated that the optimal cue integration hypothesis does not always predict the observer's perceived heading direction. Other studies support this finding of deviations from optimal cue integration hypothesis (De Winkel et al., 2015; De Winkel et al., 2010; Fetsch et al., 2012).

It is important to note that studies on optimal cue integration hypothesis did not examine circular vection but instead investigated heading. Heading research is well-suited for study on physical motion platforms (e.g., chairs moving linearly) because visual displays indicating heading match the brief motions generated by mechanical motion platforms. Moreover, the use of physical platforms to move participants or visual displays while participants are stationary, (Becker et al., 2016; Butler et al., 2015; Butler et al., 2010; Juergens et al., 2016; Kenney et al., 2021; Lishman & Lee, 1973) inevitably produces corresponding motion signals to other sensory systems. Galvanic vestibular stimulation (GVS) and CVS can stimulate the vestibular system while keeping the individual stationary, limiting other sensory systems being stimulated when examining visual-vestibular sensory integration. GVS bilaterally stimulates the mastoid processes behind each ear with an electric current that acts directly on the primary vestibular afferents.

Some studies have succeeded in producing vestibular vection using GVS (Fitzpatrick & Day, 2004; Fitzpatrick et al., 2002; Fitzpatrick & Watson, 2015; Karnath et al., 1993; St George et al., 2011). While other studies have reported that GVS does not produce a robust sense of continuous self-motion, and some studies have reported that GVS feels like tilting, or the ground suddenly moving beneath participants’ feet rather than experiencing robust self-motion (Aoyama et al., 2015; Bense et al., 2001; Dilda et al., 2014; Moore et al., 2011). Studies producing vestibular stimulation using GVS with a visual self-motion display have reported enhanced visual vection when GVS produces vestibular cues in a direction congruent with the visual direction (Cress et al., 1997), enhanced visual vection during noisy GVS (Chen et al., 2020; Cress et al., 1997; Fasold et al., 2002; Lepecq et al., 2006; Weech & Troje, 2017; Weech et al., 2018), reduced visually induced motion sickness (VIMS) and reduced cybersickness (Cevette et al., 2012; Groth et al., 2022; Sra et al., 2019; Weech et al., 2020). Further to these studies using GVS to isolate visual-vestibular interactions, their results support predictions by optimal cue integration hypothesis as they suggest that the noisy vestibular signal is down-weighted due to its unreliability, allowing visual cues to override it, enhancing vection, and reducing visual-vestibular conflict that is argued to lead to various types of motion sickness (Reason, 1978).

CVS is the process of administering cool or warm air or water via the external auditory canal, primarily stimulating the horizontal SCC. The air/water is set to be either cooler or warmer than body temperature creating a thermal gradient that changes the density of endolymph fluid in the horizontal semi-circular canal (Barany, 1906). When there is a sufficiently large difference in the endolymph fluid temperature from body temperature, a convection current produces a pressure change across the cupula. This pressure change causes the SCC's mechanoreceptors to move, producing afferent neuron firing to the 8^th^ cranial nerve, signaling to the brain that the head is rotating (Hoagland, 1932; Löwenstein & Sand, 1936). CVS has predominantly been used in clinical settings to test vestibular function by eliciting and assessing the vestibular ocular reflex (VOR) (Coats et al., 1976; Gonçalves et al., 2008; Jacobson, 1993; Sluga et al., 2021). The VOR functions as a compensatory eye movement that keeps the image steady on the retina during quick motions of the head (Britton & Arshad, 2019; Högyes, 1913). The direct link between the extra-ocular muscles and the semi-circular canals makes the VOR a reliable test of vestibular function (Goldberg et al., 1987; Högyes, 1913). CVS has also been used in biomedical engineering research to investigate fluid dynamic properties of the endolymph and semi-circular canal structures (Kassemi et al., 2005; Kassemi et al., 2004; Meiry & Young, 1967; Rabbitt, 2019; Santos et al., 2017; Steer, 1967; Wu et al., 2021). CVS has been used as an intervention for psychiatric disorders such as schizophrenia, psychosis, psychopathy (Jones & Pivik, 1985; Levy et al., 1983), has been shown to temporarily reduce spatial hemi-neglect in patients with brain injury, reduce symptoms of dementia in mice and pain in humans (Ferrè et al., 2015; Jinu et al., 2018; Moon et al., 2006). Behavioural studies have shown that individuals with impaired vestibular function are unable to accurately update their perceived position in space during CVS and real self-rotation when visual cues are not present (Karnath, 1994; Metcalfe & Gresty, 1992; Panichi et al., 2017).

CVS has also been used in neuroimaging studies to identify cortical regions associated with vestibular function (Frank et al., 2014; Frank & Greenlee, 2014; Frank et al., 2016; Klaus et al., 2020) including sensory integration mechanisms between cortical regions that integrate tactile and vestibular perception (Cedras et al., 2021) and neural correlates of somatosensory and vestibular interaction (Ferrè et al., 2012). Frank and Greenlee described a behavioral task where participants pressed a button to indicate perceived self-motion direction during caloric irrigation in an fMRI study but behavioral data were not reported (Frank & Greenlee, 2014). CVS has rarely been used to produce and measure self-motion percepts. Instead CVS has largely been used in clinical settings to study patients with vestibular deficits, canal dynamics, neural correlates of vestibular perception, and some behavioral interventions in clinical psychology.

We have identified a gap in the literature whereby few studies have used CVS to study vestibular vection and sensory integration in healthy participants. In comparison, many studies have used GVS and motion platforms, or no motion devices to investigate self-motion. An advantage of using CVS and GVS as a method to study vestibular vection and sensory integration over physical motion is that they stimulate the vestibular system without requiring physical motion, isolating the contribution of the vestibular system. An advantage of CVS over GVS and real motion is that the time course for temperature gradient in the vestibular system should stimulate the vestibular system for several seconds compared to the much shorter time courses of real motion and some GVS studies. CVS should therefore permit longer and more thorough investigations of the stimulated vestibular system and its interaction with the visual system, providing new insight into visual-vestibular sensory integration.

## Present Study

The current study examined the use of CVS to produce measurable self-motion percepts with and without conflicting visual stimuli. An air caloric irrigator was used in two experiments to induce self-rotation percepts in the vestibular system. To measure participant self-rotation in both experiments, we used a potentiometer knob device. The potentiometer knob is a circular knob that participants rotate with their index finger at the speed and in the direction in which they experience circular vection in an experimental trial. The knob indexes speed, direction and duration of vection. This method to index circular vection was presented and validated in Kirollos and Herdman's study on visual circular vection speed measurement (Kirollos & Herdman, 2021). In Experiment 1 (E1), vestibular circular vection was produced with air caloric vestibular stimulation twice in the left ear and twice in the right ear. We hypothesized that left ear cold air irrigations would induce vestibular vection in the clockwise (CW) direction and that right ear irrigations would induce vection in the counter-clockwise (CCW) direction. These hypothesized findings would be consistent with the well documented finding that cold air or water CVS relative to body temperature (> 37 °C) produces eye movements in the direction opposite to the ear being irrigated, and that warm air or water CVS (< 37 °C) produces eye movements in the same direction as the ear being stimulated (Jacobson, 1993). We also hypothesized that vestibular vection speeds and durations would not differ substantially across trials because factors assumed to impact vestibular vection speed and duration including temperature and pressure were held constant throughout the experiment (Wu et al., 2021).

The protocol in Experiment 2 (E2) was similar to E1, but a stationary visual display was presented to participants in a VR headset creating a visual-vestibular conflict. We had no specific hypotheses for E2 but we predicted three possible outcomes. The first possible outcome was that the visual cue would be used to decide self-motion. This would be consistent with visual dominance hypothesis. The second was that the vestibular cue would be used to decide self-motion. This would be consistent with vestibular dominance hypothesis. The third was that a combination of visual-vestibular cues would be used by participants to decide vection direction. This would be consistent with optimal cue integration hypothesis.

## Experiment 1

### Materials and Methods for Experiment 1: Vestibular Vection Induced by Air Caloric Vestibular Stimulation

In E1, each participant received monaural (single ear) cool air CVS in the left and right ears in four separate trials. No visual stimulus was presented to participants in this experiment. Vection experience was recorded using the potentiometer knob.

### Participants

The Carleton University Human Ethics Review Board approved the study protocol (clearance #104652). Participant signed the approved consent form before the experiment began. Twenty-five university students participated in the experiment in exchange for course credit. Seventeen participants were female, and two participants were left-handed (*M*_AGE _= 22.7, *SD*_AGE _= 5.6). No participants reported a history of vestibular problems. One participant did not complete the study, leaving 24 participants in the final analysis.

### Apparatus and Measures

#### ICS NCA 200 Air Caloric Irrigator

**Figure 1.**
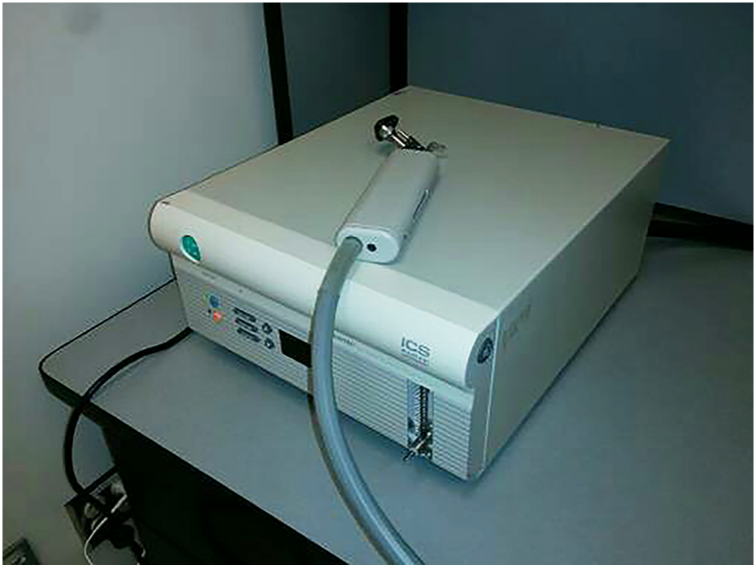
ICS air caloric irrigator.

The irrigator pictured in Figure 1 generated air flow at 18°C at 10 liters per minute. These values were chosen to maximize chances of inducing vestibular vection. A hose attachment delivered air via a glass speculum fitted with a disposable rubber tip to one of the participant's ears depending on experiment counter-balance order.

#### Control Knob

**Figure 2.**
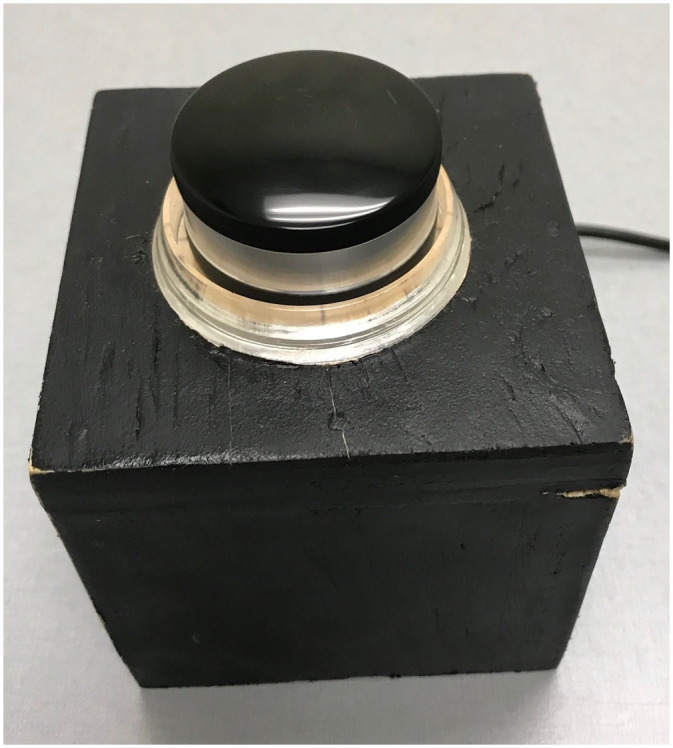
Potentiometer knob used by participants to index vection.

The Spintrak rotary knob in Figure 2 was used to index speed, direction and duration of circular vection (Kirollos & Herdman, 2021). The circular knob had a 4.4 cm diameter and could be turned CW or CCW indefinitely. It had a tachometer and a high-resolution pulse rate of 1200 units over 360° for precise knob position tracking and recording. The knob was USB-integrated with custom software that logged turn rates in °/s at 75 Hz. It was housed in a custom-built wooden box and rested on the participant's stomach during testing.

#### Computer

The computer logging knob data had an Intel Core i7 processor, an NVIDIA GeForce GTX980 graphics card and 16 GB RAM.

#### Stimuli

CVS trials were all performed at the same and constant temperature of 18°C and constant air pressure of 10 liters/min in all trials. Participants underwent two left-ear and two right-ear irrigations, totaling four trials per participant in the experiment. Trial direction data was analyzed on a per trial basis to determine whether irrigated ear would result in predicted vection direction. Each irrigation lasted from 90 s to a maximum of 180 s depending on when the participant reported feeling a robust spinning sensation in either the CW or CCW direction.

#### Procedure

Participants were tested individually in a quiet room with eyes closed and wore a blindfold. They lay down in the supine posture on a table with their heads rested on a 30°-angle wedge pillow to optimally stimulate the horizontal SCC (Baloh, 2003). Participants rested the potentiometer knob on their stomach and had their dominant hand's index finger positioned on the knob. They held the knob with their non-dominant hand. The experimenter visually inspected the participants’ ear for any obstruction that may reduce efficacy of CVS before the experiment began. Participants were asked to demonstrate CW and CCW rotation of the knob with their dominant index finger to avoid confusion about direction during trial response periods. When irrigation began, participants could describe self-motion direction and strength to the experimenter. The trial was terminated by the experimenter if no robust spinning was reported within 180 s. Trial order was randomized such that equal numbers of participants began the experiment with left ear irrigations as right ear irrigations. Remaining irrigations alternated from left to right or right to left. If and when participants verbally reported a robust spinning sensation, the experimenter removed the irrigator hose from the participants’ ear. The participant was instructed to rotate the potentiometer knob in the direction and at the speed they felt circular vection while participants’ eyes remained closed until circular vection ceased. Participants were instructed to verbally report when they no longer experienced vection. The experimenter ended the trial once participants verbally reported that the spinning sensation had stopped. If participants reported swaying, rotation but with no precise direction, or no self-rotation, they were instructed not to rotate the knob.

Participants were given at least 10-min breaks to allow time for endolymph fluid in the inner-ear to reach normal body temperature and resulting vestibular circular vection to subside before the subsequent trial began. During breaks, participants kept their eyes closed for the first two minutes to avoid any possible nausea and disorientation resulting from any lingering self-motion percepts. This procedure was repeated four times, alternating left and right ear CVS. The experiment lasted approximately 90 min.

### Results

Cold irrigation generally results in reflexive beating of the eye in the direction opposite to the irrigated ear (Jacobson, 1993). This means that cold irrigations in the left ear should have resulted in left-to-right eye movements and that right-ear irrigation should have resulted in right-to-left eye movements. Vection direction data were analyzed with a McNemar test on a per-trial basis. Vection speed and duration data were analyzed using two-tailed within-subjects t-tests to compare left ear to right ear trials. All data were analyzed in SPSS version 25.0.

#### Vection Direction

**Figure 3.**
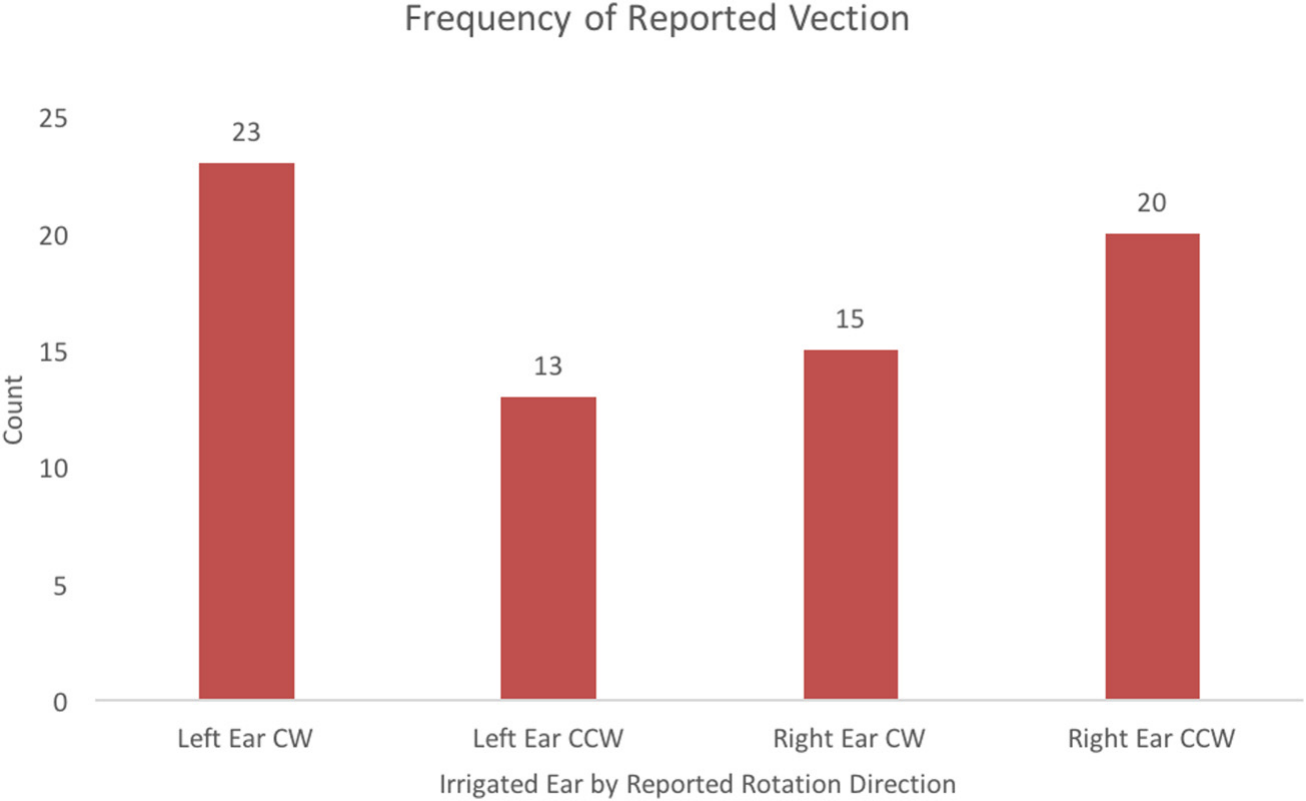
Frequencies of reported vection direction based on the ear that was irrigated.

Vection was experienced on 71 of 96 trials (74%) across all participants. Of the 48 left-ear irrigation trials, participants reported experiencing vection in the CW direction 23 times and in the CCW direction 13 times. Participants did not report vection in the 12-remaining left-ear caloric irrigation trials. Of the 48 right-ear irrigation trials, participants reported vection in the CW direction 15 times and in the CCW direction 20 times. Participants did not report vection in 13 right-ear irrigation trials. These data are summarized in Figure 3. The McNemar test compared proportions of vection direction based on ear irrigated and showed a significant difference for perceived direction based on ear irrigated, *p* = .008.

#### Vection Speed

Mean speed of vection (°/s) for the duration of the trial when participants reported vection was compared across left ear (*M* = 112.2°/s) vs. right ear (*M* = 105.5°/s) trials using a within-subjects t-test. The effect of ear irrigated (left or right) on speed of vection was not significant, (*t *< 1, *df *= 20) as predicted.

#### Vection Duration

Vection duration was calculated as the cumulative amount of time (s) within a trial that a participant rotated the knob. A within subjects t-test comparing vection resulting from left ear irrigations (*M* = 54.7 s) to vection from right ear irrigations (55.3 s) revealed that effect of ear irrigated on vection duration was not significant (*t *< 1, *df *= 20) as predicted.

E1 revealed that cool air caloric irrigation can produce robust self-rotation percepts in a 74% of trials when participants had their eyes closed. Results indicated there was a small but significant bias for vection to be perceived in the predicted direction in the CW and CCW directions based on findings from the McNemar test. These findings are not as clear as expected but are generally consistent with our prediction. E2 built on this finding by exploring how a visual conflict can impact vestibular vection measures.

## Experiment 2

### Materials and Method for Experiment 2: Vection Induced by air Caloric Vestibular Stimulation While Viewing a Stationary Display in a VR Headset

In E2, participants underwent similar protocol as in E1 but observed a black and white vertical stripe pattern representing the interior of a virtual optokinetic drum in a VR headset immediately after caloric stimulation. This is in contrast to E1 when participants kept their eyes closed throughout the trial. A VR headset was used in this experiment in place of a physical optokinetic drum because VR headsets can be easily used in different body orientations and head poses. Because the horizontal SCCs are optimally stimulated with CVS when the head is pitched forward 30° from Earth's horizontal, the experimenter can easily set the stripes of the virtual optokinetic drum to be in line with the participants’ longitudinal axis while laying supine. Moreover, self-motion perception in synthetic environments has increasingly practical applications for gaming, entertainment, training and simulation across many domains. The black and white vertical stripe pattern in E2 was stationary and was not predicted to produce self-motion percepts in participants. The combination of CVS signaling vestibular vection with the stationary display that did not signal visual vection was designed to create a visual-vestibular conflict. The purpose of this experiment was to measure vection speed, direction and duration using the potentiometer knob during the presented visual-vestibular conflict.

### Participants

A total of 25 undergraduate students participated in this experiment in exchange for course credit. All participants in E2 were different than those in E1. Two participants did not complete the experiment, leaving 23 participants in the final analysis (*M*_AGE _= 22.6, *SD*_AGE_ = 8.0). Six participants were male and three were left-handed. None of the participants reported any vestibular disorders.

### Apparatus and Measures

#### VR Headset

An Oculus Rift DK2 VR headset provided a 110° diagonal visual angle, a native resolution of 960 × 1080 pixels per eye, and a 75 Hz refresh rate. The left and right eye displays presented the same image at different perspectives permitting 3D perception of the stimuli. All other apparatus including the knob, caloric irrigator, and computer were the same as in E1.

#### Stimuli

Air caloric vestibular irrigations were all performed at a constant temperature (18°C) and with constant air pressure (10 liters/min) in all trials. Participants underwent two left-ear and two right-ear irrigations, totaling four trials per participant in the experiment. The graphical display representing the optokinetic drum was built in Unreal Engine version 4.8. Participants were not notified that the display was stationary. The display subtended 110° of the observers’ visual field. The virtual drum had a 200-cm diameter and the observer viewed the drum from 100 cm, virtually positioned in the center of the drum. Each stripe in the display corresponded to a width of 33 cm in the virtual graphics environment, and subtended a horizontal visual angle of 10.85° at the virtual viewing distance of 100 cm.

#### Procedure

Participants lay down in the supine posture on a table with their heads rested on a 30°-angle wedge pillow to optimally stimulate the horizontal SCCs. Participants received caloric irrigation while wearing the headset for 90–180 s. Participants had their eyes closed during irrigation and no image was displayed on the headset. When irrigation ended participants were prompted to open their eyes and the striped pattern was displayed in the VR headset. Participants were instructed to look at the display and rotate the knob at the speed and in the direction that they experienced vection and not to rotate the knob if they did not experience clear vection. Participants stopped rotating the knob when they no longer experienced vection, closed their eyes, and notified the experimenter, ending the trial. Participants underwent two left-ear and two right-ear irrigation trials with alternating ear irrigations. Breaks were administered under the same protocol in E1 and the experiment lasted approximately 90 min.

### Results

The experimental design was identical to E1 wherein direction data was analyzed on a per trial basis using a McNemar test and speed and duration data were analyzed in a one-way repeated measures design comparing data for left to right ear irrigations across all trials using a within-subjects t-test. All data were analyzed in SPSS version 25.0.

#### Vection Direction

**Figure 4.**
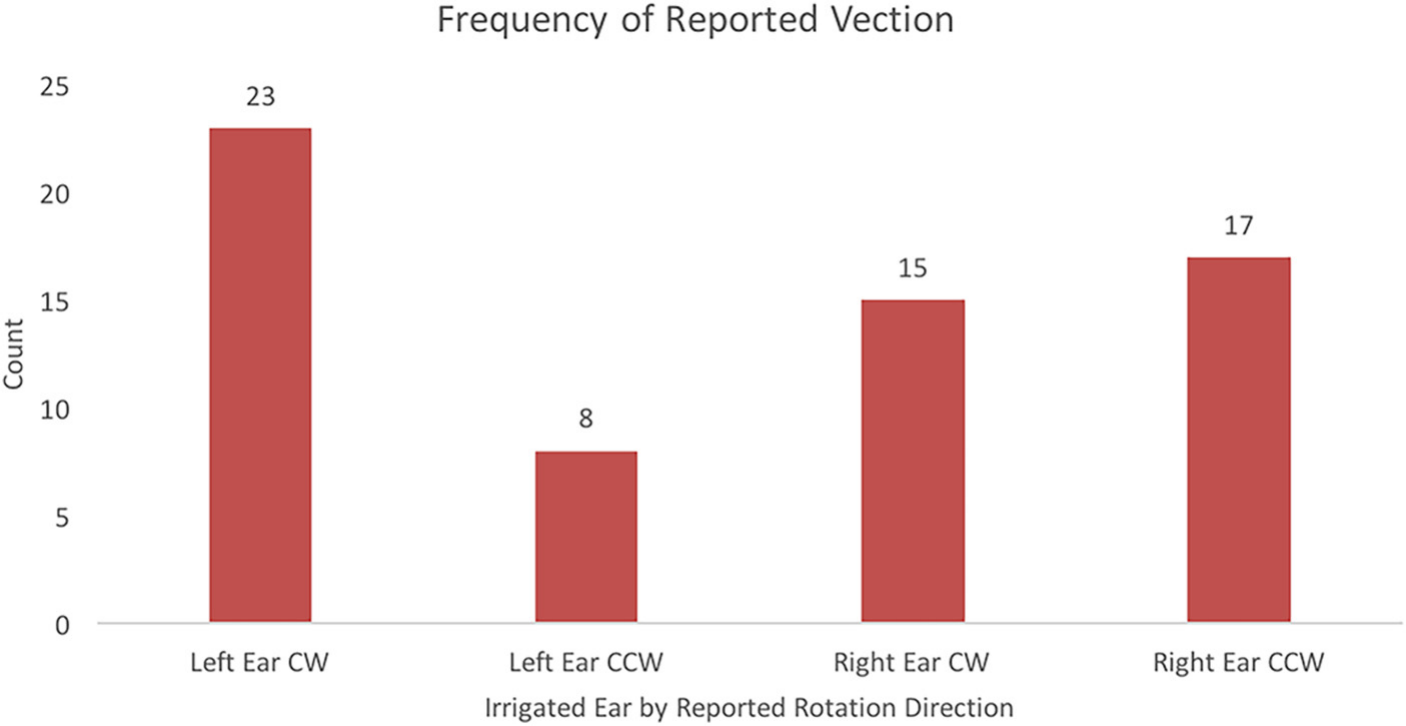
Frequencies of reported vection direction based on the ear that was irrigated while viewing a stationary drum pattern.

Vection was experienced in 63 of 92 trials (68%). A McNemar test was used to analyze vection direction data based on ear irrigated. Data used in the McNemar test are presented in Figure 4. Results from the McNemar test showed a significant difference for ear irrigated on direction of perceived circular vection, *p = *0*.*008. Of the 46 left-ear irrigation trials, participants reported experiencing CW vection on 23 trials and reported CCW vection on 8 trials. Of the 46 right-ear irrigation trials, participants reported experiencing CW vection on 15 trials and reported CCW vection on 17 trials. Participants did not experience vection on 15 left-ear and 14 right-ear trials.

#### Vection Speed

Data for knob rotation speed were compared for left (M = 78.8 °/s) vs. right ears (M = 86.3°/s) in a within subjects t-test. There was no difference in average vection speed reported in left vs. right ear, *t*(21) < 1.

#### Vection Duration

Data for vection duration was compared for left (44.6 s) and right (45 s) ears in a within subjects t-test. There was no significant difference for vection duration on irrigated ear (*t *< 1, *df *= 21).

#### Comparing Vection Speed and Duration in Experiments 1 and 2

To examine how visual-vestibular conflict impacts vection generated from CVS, mixed-factor ANOVAs were used to analyze speed and duration across E1 (eyes closed: no visual-vestibular conflict) compared to E2 (eyes-open: visual-vestibular conflict). The within-subjects factor in the mixed-factor ANOVAs was ear irrigated (left vs. right ear). The between-subjects factor in the mixed-factor ANOVAs was Experiment (E1: eyes closed vs. E2: eyes open).

**Figure 5.**
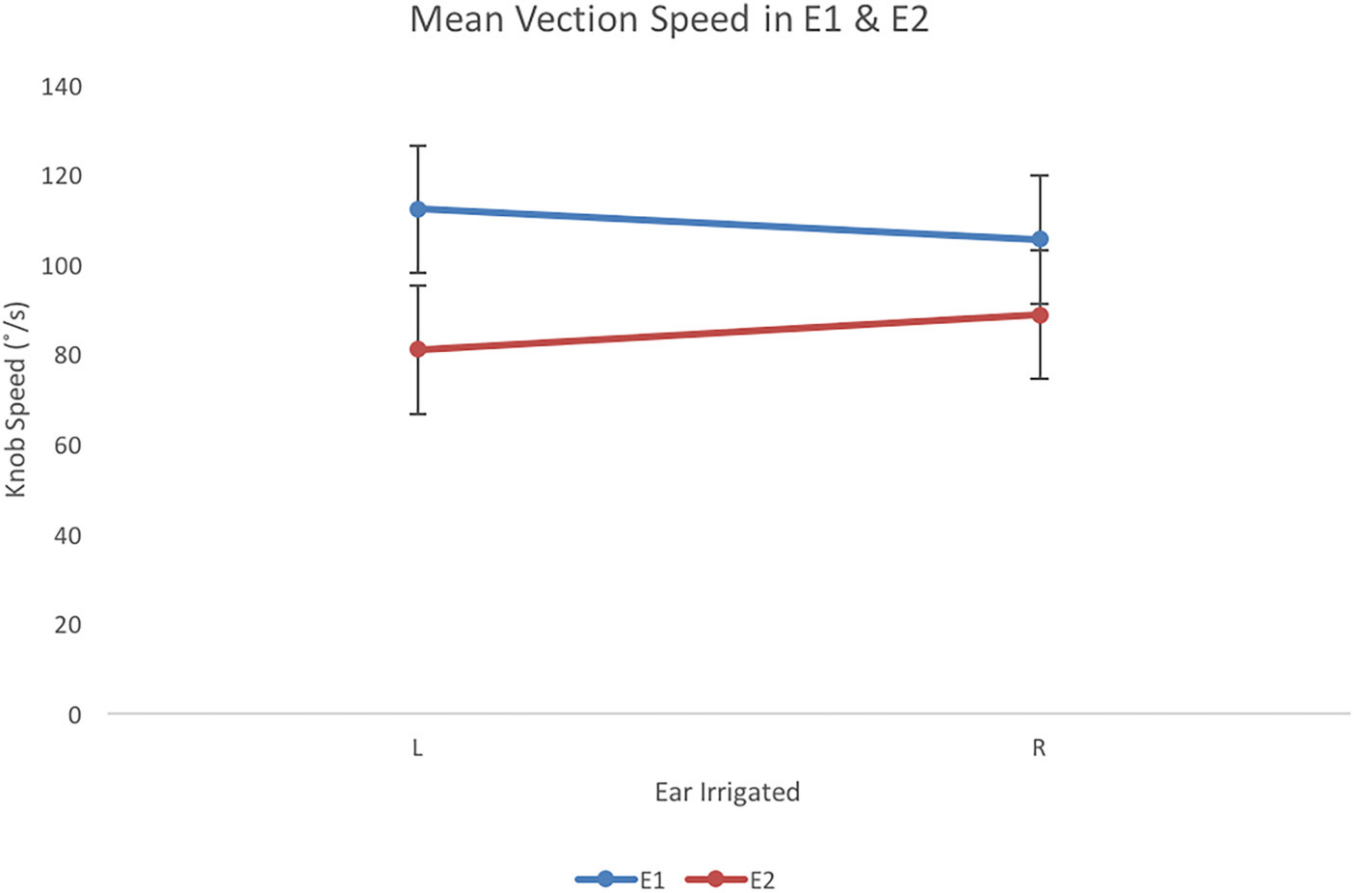
Vection speed (°/s) for left (L) and right (R) ear irrigations for E1 and E2. Error bars represent 95% confidence intervals for the analysis.

**Figure 6.**
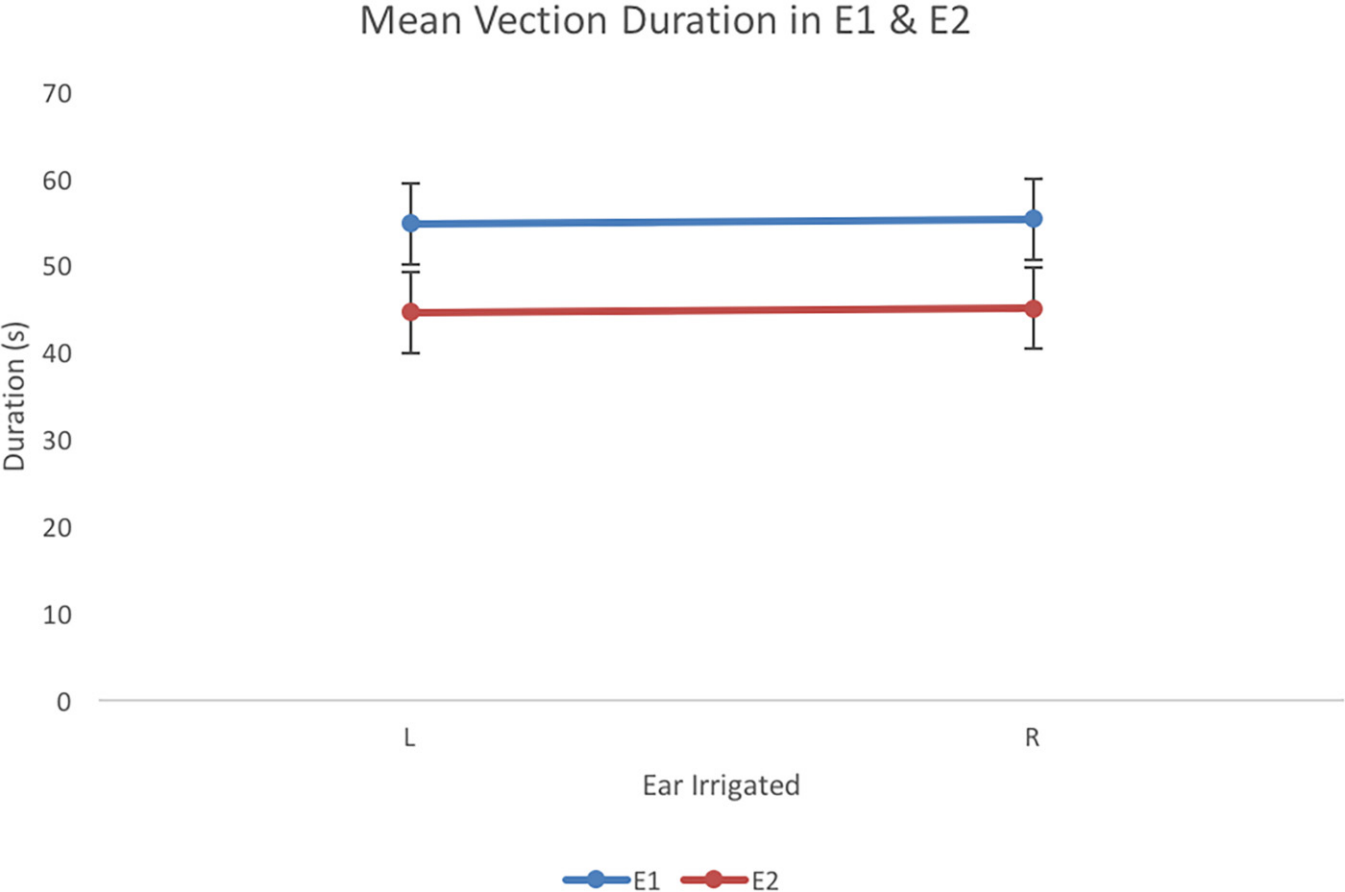
Vection duration (s) for left (L) and right (R) ear irrigations for E1and E2. Error bars represent 95% confidence intervals for the analysis.

There was a significant effect of Experiment on speed, *F*(1, 41) = 51.36, *p* < .001, *R*^2 ^= .56 and on duration, *F*(1, 41) = 158, *p* < .001, *R*^2 ^= .79. Findings comparing speed across experiments and duration across experiments are presented in Figures 5 and 6, respectively. Vection was faster and longer when participants did not observe a visual display (E1) compared to when participants attended to a visual display (E2). There were no significant differences in ear irrigated, nor any interactions (all *F*s < 1, *df* = 1, 41).

## Discussion

### Discussion of Experiment 1 Results

The first goal of this paper was to determine if vestibular circular vection can be induced and measured using CVS. The second goal of this paper was to determine whether a conflicting visual display signaling no vection would impact vestibular vection.

In E1, CVS produced vection on 74% of trials indicating that vestibular vection can be produced with CVS and recorded by the potentiometer knob. Vection lasted roughly 55 s. This is much longer than the transient self-motion produced during some studies using GVS or with the use of physical motion cueing platforms. The long vection periods produced by CVS in E1 will allow researchers to thoroughly investigate vestibular percepts by pairing caloric irrigation with other stimuli such as visual displays, and auditory stimuli to study sensory integration.

Cold irrigation generally results in reflexive beating of the eye in the direction opposite to the irrigated ear (Jacobson, 1993). This means that cold irrigations in the left ear should have resulted in left-to-right eye movements and that right-ear irrigation should have resulted in right-to-left eye movements. Left-to-right eye movements are consistent with eye movements that would compensate for CW spinning. Right-to-left eye movements are consistent with eye movements that would compensate for CCW spinning. Vection directions from E1 are consistent with clinically observed nystagmus direction patterns reported by Jacobson and Newman, and are consistent with our first hypothsis (Jacobson, 1993). In some trials, participants reported CCW vection when the left ear was irrigated, CW vection when the right ear was irrigated and sometimes no vection which is opposite to the observed general pattern of results reported by Jacobson and Newman. Monaural air caloric stimulation used in this study may have caused a weak vestibular signal resulting in variability in self-rotation direction reports in some trials. Because one ear was irrigated, the endolymph fluid properties in the irrigated ear changed, but not in the other. During real spinning, the mechanoreceptors in both ears signal reciprocal motion, providing stronger and clearer self-motion direction information. Thus, it may be that monaural air CVS used in the present study was not sufficient to produce a robust spinning sensation in the expected direction in all trials, creating variability in perceived self-rotation direction.

There was no significant difference of ear irrigated on vection speed and vection duration. This was expected as temperature and airflow pressure were the two variables predicted to mediate vection speed and duration and were held constant in E1, consistent with our second hypothesis.

### Discussion of Experiment 2 Results and Comparisons to Experiment 1 Results

In E2, participants viewed a visual display signaling no self-motion in a VR headset while experiencing vestibular vection from CVS. Results showed that vection was experienced despite the presented visual-vestibular conflict. Vection direction showed the same general pattern of results in E2 as in E1: left ear irrigation resulted in CW vection and right ear irrigation resulted in CCW vection. However, in E2 vection speed was significantly slower and duration significantly shorter compared to E1. Results from E2 suggest that the visual display signaling no self-motion provides conflicting sensory input that significantly reduced vection but that vestibular vection was still experienced.

Findings from E2 support optimal cue integration hypothesis. Optimal cue integration best explains these results in so far as it predicts that visual and vestibular systems do not dominate during conflict. Instead, self-rotation experience and its direction will be determined by the most reliable cue based on Bayesian *a posteriori* probability modeling (Ernst & Banks, 2002; Knill & Pouget, 2004). “Reliability” in optimal cue integration hypothesis is defined as the inverse of a cue's variability (Fetsch et al., 2010; Fetsch et al., 2009). Under optimal cue integration hypothesis, it is assumed that cue reliability for the visual and vestibular cues in E2 were the same because they were not manipulated and were presented in a way that was consistent across trials. Therefore, the visual and vestibular cues were thought to have similar reliabilities that resulted in reduced vestibular vection accordingly. If in E2, the visual system dominated during the conflict, no or negligible vection (i.e., speed and duration of vection would be near 0) would have been reported as this would indicate that the signals form the visual cue overrode the vestibular cue. In contrast, if findings supported vestibular dominance hypothesis, vection speed and duration results in E2 would have been very similar to results in E1. Whereas we found a significant difference in vection speed and duration in E2 compared to E1. Therefore, E2 results do not support visual dominance nor vestibular dominance.

Findings of reduced vection speed and duration during E2 compared to E1 can also be explained by VOR suppression when the eyes were open in E2 (Cullen, 2012; Reinhardt-Rutland, 1988). The VOR is known to last longer when there is no visual target to fixate than when there is a visual target to fixate (Cullen, 2012; Riecke, 2011) . In E1, the VOR presumably lasted longer than in E2 because the eyes were closed in E1. Findings from E2 provide behavioral data consistent with findings of suppressed VOR during visual fixation than without as they show that vection is reduced when eyes are fixating a visual display compared to when they are closed.

In related visual-vestibular sensory integration research, Lepecq et al. have shown that perceived self-motion trajectory can be altered when a visual display is presented simultaneously with GVS that induces the perception of tilting compared to a visual-only control condition (Lepecq et al., 2006). Generally, visual vection takes several seconds to experience, however, research by some researchers has shown that visual vection latency can be reduced when noisy GVS (i.e., GVS signaling motion in no particular direction) is administered while viewing a visual display signaling vection (Weech, 2017; Weech & Troje, 2017). Additionally, Weech, Varghese, and Barnett-Cowan, have shown that visual vection is stronger with the use of noisy vestibular signals (Weech et al., 2018). Specifically, the authors leverage the use of an unreliable vestibular signal causing sensory reweighting to favour the visual cue, consistent with predictions made by optimal cue integration hypotheses. Cress et al. (1997) reported significantly more convincing motion by participants when GVS that induced roll axis tilt was combined with a visual display indicating tilt in the same direction compared to only viewing the visual display. Taken together, these studies indicate that vestibular cues for self-motion perception can alter visual vection perception when they are presented simultaneously by manipulating the reliability of the vestibular cue. Findings from E2 build on results from these studies and contribute to our understanding of optimal cue integration hypothesis by demonstrating that vestibular cues presented using caloric irrigation with visual cues also alter vection by reducing vection speed and duration when a visual stimulus signaling no self-motion is presented.

Gallagher et al. (2020) showed that sensitivity to the vestibular cue is reduced when a visual display is paired with an inconsistent galvanic signal on the same axis of rotation but in a different direction. The present study extends these findings as we demonstrate that vestibular vection speed and duration is reduced during incongruent visual-vestibular displays. Our findings together with those of Gallagher and colleagues show a link between reduced vestibular sensitivity and reduced vestibular vection speed and duration during conflicting visual-vestibular presentations.

## Conclusions & Future Work

In this paper, we set out to determine if vestibular circular vection can be induced and measured using caloric vestibular stimulation and to determine whether a conflicting visual display signaling no vection would impact vestibular vection. In the current study, CVS resulted in measurable vestibular vection when there was no conflict (E1) and reduced but compelling vestibular vection during a conflicting visual display signaling no self-motion (E2). Long vestibular vection durations were obtained despite the conflict presented in E2. Results from this experiment provide support for optimal cue integration hypothesis by demonstrating that vestibular cues still produced vection despite visual cues impairing vection. Therefore, both visual and vestibular cues influenced vection perception.

Based on the current study, using CVS with visual presentations in VR headsets appears to be a promising method to investigate visual-vestibular sensory integration. The present study established a method for conducting future investigations of visual-vestibular integration using moving visual cues by generating visual cues in a VR headset simultaneously with a vestibular self-rotation cue using CVS. Future research should track and record eye movements associated with CVS and compare them to the data from the potentiometer knob to understand the relationship between the VOR and vestibular vection. Further research can investigate how varying features of visual optic flow trajectories such as speed and direction presented in combination with CVS impacts vestibular vection. Future research can also investigate how varying temperature and pressure of the caloric stimulus can impact vestibular vection measures indexed when using the potentiometer knob used herein. Moreover, future research can combine the use of CVS with GVS and/or motion bases to expand the range of realistic vestibular cues beyond those provided by CVS. Future research can replicate this study in a physical optokinetic drum to examine if there are any differences in findings between virtual and physical optokinetic drum. Finally, future studies can employ the potentiometer knob from the current study to measure vestibular vection from CVS to compare the difference in perceived vection speed, direction and duration between participants with vestibular damage to those with no vestibular damage to investigate self-motion perception differences.
